# Environmental Impact and Relative Invasiveness of Free-Roaming Domestic Carnivores—a North American Survey of Governmental Agencies

**DOI:** 10.3390/ani7100078

**Published:** 2017-10-14

**Authors:** Ana Lepe, Valerie Kaplan, Alirio Arreaza, Robert Szpanderfer, David Bristol, M. Scott Sinclair

**Affiliations:** 1 SeaSearch Biological Surveys, 1275 S. Lee Street, St. David, AZ 85630, USA; ana.lepe@outlook.com (A.L.); shinyhappygoth@gmail.com (V.K.); alirio.arreaza@gmail.com (A.A.); rszpand@outlook.com (R.S.); 2 Statistical Consulting Services, Winston-Salem, NC 27127, USA; david@statistical-consulting-services.com

**Keywords:** cat, dog, domestic carnivore, ferret, environmental impact, government, invasive, non-native species, North America, survey

## Abstract

**Simple Summary:**

This paper reports on a survey that explores the impact of three non-native domestic carnivores—dogs, cats, and ferrets—on the native wildlife of the United States (US) and Canada. Government agencies were asked to document the number and frequency of sightings, and the degree of concern resulting from free-roaming animals on urban, suburban, rural, recreational areas, and wildlands in their jurisdictions. Results confirm the existence of free-roaming cats and dogs throughout North America, as well as their profound impact on native wildlife, with cats being the major offenders. Except for an occasional stray, free-roaming ferrets were “never” or “rarely seen”; no agency reported that ferrets caused environmental harm. This is the first study to compare the relative impact of free-roaming dogs, cats, and ferrets. It shows differences in how these three animals react to novel environments. For the US and Canada, free roaming cats and dogs meet the definition of an “invasive” species, whereas ferrets do not. However, the way we as a society view these animals, our attitudes and perceptions, may influence how governmental agencies manage and control them.

**Abstract:**

A survey of the United States and Canadian governmental agencies investigated the environmental impact and relative invasiveness of free-roaming domestic non-native carnivores—dogs, cats, and ferrets. Agencies represented wildlife, fish, game, natural or environmental resources, parks and recreation, veterinary and human health, animal control, and agriculture. Respondents were asked to document the number and frequency of sightings of unconfined animals, evidence for environmental harm, and the resulting “degree of concern” in their respective jurisdictions. Results confirmed the existence of feral (breeding) cats and dogs, documenting high levels of concern regarding the impact of these animals on both continental and surrounding insular habitats. Except for occasional strays, no free-roaming or feral ferrets were reported; nor were there reports of ferrets impacting native wildlife, including ground-nesting birds, or sensitive species. This is the first study to report the relative impact of free-roaming domestic carnivores. Dogs and cats meet the current definition of “invasive” species, whereas ferrets do not. Differences in how each species impacts the North American environment highlights the complex interaction between non-native species and their environment. Public attitudes and perceptions regarding these species may be a factor in their control and agency management priorities.

## 1. Introduction

Concern over the impact of non-native species on novel ecosystems has been a major focus of both conservationists and governmental agencies world-wide, which has resulted in the mandated monitoring and management of non-native species [1]. Historically, humans migrating to new geographic locations brought their animals and plants with them, resulting in large-scale introductions of non-native species to novel ecosystems. These activities created both competition with and, in many cases, destruction of, native species and habitats. Concepts of “nativeness” and “alien” species did not arise, however, until the 19th century, later engendering a global discussion of “biologic invasions” exemplified by Charles Elton’s 1958 treatise, *The Ecology of Invasion by Animals and Plants* [2,3]. Currently an “invasive” species is defined as “any species not native to the ecosystem likely to cause economic or environmental harm, or harms human health” [4]. Today, Canada and the United States are among the nations to develop positions regarding “invasive” species, along with mandates to control them [4,5].

With the exception of the Alaskan malamute, the domestic carnivores—the dog (*Canis lupus familiaris*), cat (*Felis silvestris catus*), and ferret (*Mustela putorius furo*)—each meet the definition of “nonnative” species [6,7,8,9,10,11]. The ferret (also “European” ferret) should not be confused with the wild black-footed ferret (*Mustela nigripes*), one of North America’s most endangered mammals [12].

In 1993, a US Congressional report “*Harmful Non-Indigenous Species in the United States*” named feral dogs and cats as “two of the three most common subjects of wildlife control efforts” of US national parks and wildlife reserves; the ferret was not mentioned [13]. In the US alone, cats reportedly kill 1.3–4.0 billion birds and 6.3–22.3 billion mammals annually, resulting in economic damages around $14 billion (USD) [14,15,16,17,18,19,20,21,22,23,24]. All 50 states report the existence of feral dogs, with resultant damage to both natural and agricultural resources estimated at more than $620 million annually [21,22,25]. In a 10-year review by Bergman et al., the impact from feral dogs was seen not only in their predation, but also in their causing behavioral changes in both wildlife and livestock, and their role in disease transmission to other animals and to humans [25].

Although it is legal to own a dog or cat throughout the North American continent and the surrounding islands, some jurisdictions prohibit ferrets. These include the states of Hawaii and California, and certain cities [26,27,28,29]. As a basis for restricting the ferret, government agencies cite reports from New Zealand and other island nations where European settlers released thousands of ferrets, cats, and other domestic species in the late 19th century resulting in a disruption of local ecosystems [30,31]. In response to a Citizen Petition to California regarding its ban on ferrets, a nation-wide survey was conducted in 1996–1997 by the California Department of Fish and Game Habitat Conservation and Planning Branch. Although state agencies reported urban sightings of “stray” ferrets as “none” (15–30%), or “rare or “sporadic” (28–56%), and no state suspected or documented “breeding” (feral) ferrets, California continued its ban on domestic ferrets [32].

SeaSearch Biological Surveys (SeaSearch), became interested in the discrepancy in regulatory response. Therefore, the purpose of this study was to examine the evidence for invasiveness from the three domestic carnivores using a survey to assess their relative levels of impact on the North American environment and agriculture. The survey was designed to document the existence of free-roaming (“unconfined”) animals—those outside of the direct control of humans, and the evidence for “harm” caused to native wildlife and agricultural species. It also elicited the “degree of concern” generated by the presence and impact of these three animals when in the unconfined state. This article focuses on the impact to native wildlife and the environment, including parks and recreational areas. The impact on agriculture is being reported separately.

## 2. Materials and Methods

State agencies of the US (including Washington, DC) and provincial agencies of Canada were selected that had roles in the control, management, or impact of domestic or wild animals. Agencies represented natural or environmental resources, fish, game and wildlife, agriculture, parks and recreational areas, and human and veterinary health departments (e.g., “State Veterinarian”), where such a department or position existed. Due to California’s statewide ban on ferrets, both the state and county agencies were queried. County agencies represented parks and recreation, health, agriculture, and animal control.

The survey was disseminated in the form of a questionnaire (see Supplementary Materials Figure S1). It addressed agency and responder demographics; terminology used by the agencies to classify dogs, cats and ferrets (e.g., “house-pet/companion”, “domestic/domesticated”, “exotic”, “wild”–”wildlife”, etc.); “sightings” and “existence” of unconfined animals”; “frequency of unconfined animal sightings” and estimates of “number of animals” at each sighting; “effects (impact)” on wildlife, parks-recreational areas, and agriculture; and, “actions taken” (e.g., “none”, “live trap”, “refer to another agency”, etc.). Rating systems varied depending on the question. Responses requiring estimates were presented in a discrete ratings system to reduce open-ended responses and decrease the response variability. Each question included a “comments” section to allow for clarification or documentation of the ratings given. Respondents were also asked to rank their “degree of concern” and to provide any “special concerns” regarding unconfined animals in urban, rural-agriculture, parks-recreational, and wildlands-undeveloped settings.

Initial agency contacts were identified using the Internet. When no comparable agency was found, the highest jurisdictional level was contacted by telephone in an attempt to identify the responsible organizational unit or individual. Using a script, potential respondents were then contacted by telephone to invite their participation.

In the initial telephone calls, participants were asked how they wished to receive the survey. Based on their requests, the survey was distributed to agencies in electronic format (.pdf—Adobe Acrobat^®^), by facsimile, or mailed in “hard-copy”. Delivery, receipt, and correspondence were tracked and recorded. Completed surveys and correspondence were saved in or converted to electronic media. At least six attempts were made to reach non-responding jurisdictions, and results of these efforts were recorded. Data collection included both coded ratings and text. All data were periodically updated and maintained in a database (Microsoft Access^®^). Analyses were performed on individual agency categories, and in combination with other agency categories.

Responses were categorized as a “completed survey” (S) or a “nonsurvey response” (NS), when some information responsive to the questionnaire was provided. Failure or refusal to respond in the absence of any responsive information was recorded as a “nonresponse” (NR). For NRs, the next organizational level was contacted to determine whether another individual or organizational unit would be more appropriate. Results reported herein reflect all responding state or provincial agencies, with a separate set of analyses performed on the combined CA county agencies.

The Mann-Whitney test was used to compare responses for dogs, cats, and ferrets, with respect to frequency of sightings (0 = “Never”, 1 = “Historical”, 2 = “Rare”, 3 = “Common”, or 4 = “Frequent”), degree of concern regarding sightings (−2 = “Definite Concern”, −1 = “Some Concern”, 0 = “No Concern”, 1 = “Some Benefit”, or 2 = “Definite Benefit”), and the number of animals per sighting. Means and standard error of the mean (sem) are presented for each comparison. Pairwise comparisons are designated as ‘a’: dogs vs. cats, ‘b’: ferrets vs. dogs, and ‘c’: ferrets vs. cats. *p*-values between 0.0001 and 0.001 are presented as “*p* < 0.001”, *p*-values between 0.001 and 0.01 are presented as “*p* < 0.01”, *p*-values between 0.01 and 0.05 are presented as “*p* <0.05”, and *p*-values exceeding 0.05 presented as “ns”.

## 3. Results

Responses were received from 96.7% (59 of 61) jurisdictions at the state/provincial level, representing 108 agencies (85 S; 23 NS). The majority of responding agencies (S + NS) were wildlife, fish, game, natural or environmental resources (58; 53.7%), with the remaining from parks and recreation (13; 12.0%), State Veterinarian (15; 13.9%), health (12; 11.1%), and agriculture (10; 9.3%). Included among the NS were those claiming “no data” (10.3%), or that they were not the “correct” agency but gave referrals to other agencies (8.3%). One hundred sixty-four agencies were NRs; no agency responded from Arizona and Kansas. When reasons were given, NRs stated that they did not wish to participate, lacked the funding or time to participate, or refused to participate unless they knew more about the surveyor.

Demographics showed that respondents represented a wide range of organizational levels and expertise. Of the 69 US agencies providing sufficient organizational information, 29.0% were agency or department heads (level 1), 30.4% were supervisors (level 2), and 40.6% had no supervisory responsibility (level 3). Of the 10 Canadian provincial agency responders, 18.2% were level 1, 18.2 % level 2, and 63.6% level 3.

Thirty-five (60.34%) of California’s 58 counties responded. Not all counties had agencies comparable to those at the state level, and no county agency represented wildlife, fish, game, natural, or environmental resources. Instead of a veterinary unit, most counties had an animal control division. Forty-five county agencies responded (S + NS): 36 S (80.0%) and 9 NS (20.0%). Of responders (S + NS), 23 (51.1%) were animal control, 6 (13.3%)-parks (beaches)-recreation, 12 (26.7%)-agriculture, and 4 (8.9%)-health departments. One hundred forty-eight county agencies were NR, which either failed or refused to respond or provided no information responsive to the survey. Of the 31 counties completing the demographics section, 38.7% were level 1 individuals, 41.9% level 2, and 19.3% level 3.

### 3.1. Sightings of Unconfined Animals

Respondents were asked to estimate the “frequency of sighting” of unconfined dogs, cats, and ferrets, the number of animals seen at each sighting, and “degree of concern” regarding unconfined animals in the following areas: urban-suburban; rural-agricultural; parks-recreational areas; wildlands; and, the existence of “free-living (surviving > 1 week)”, “feral (breeding)”, or “naturalized” animals.

**Figure 1:**
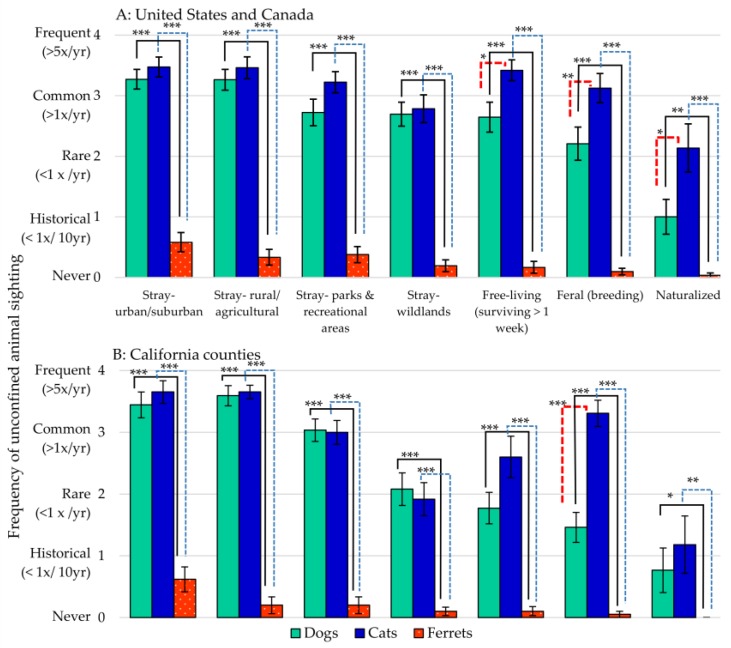
Frequency of sighting for unconfined dogs, cats, and ferrets for: (**A**) United States, and Canada (**B**) California counties. Rating mean ± standard error of the mean (sem). Asterisk represents significant difference; * *p* < 0.05, ** *p* < 0.01, *** *p* < 0.001.

The “frequency of sightings” of an unconfined dog, cat, or ferret was reported covering specified time periods (Figure 1). In most areas, sightings of “stray” dogs and cats were rated “common (>1 time per year)”, whereas ferret sightings were rated as either “historical” (“not in the past 10 years”) or “never”. Unconfined cats were sighted more frequently than dogs in most locations; however, both species had ratings for “commonly seen (>1 time per year)”, “free-living”, “feral”, or “naturalized” animals. Similar results were reported from responding California county agencies. Unconfined cats the most frequently observed animal, except for a slight majority of dogs spotted in parks and recreational areas, and in wildlands. No county agency reported “free-living”, “feral”, or “naturalized” ferrets.

**Figure 2:**
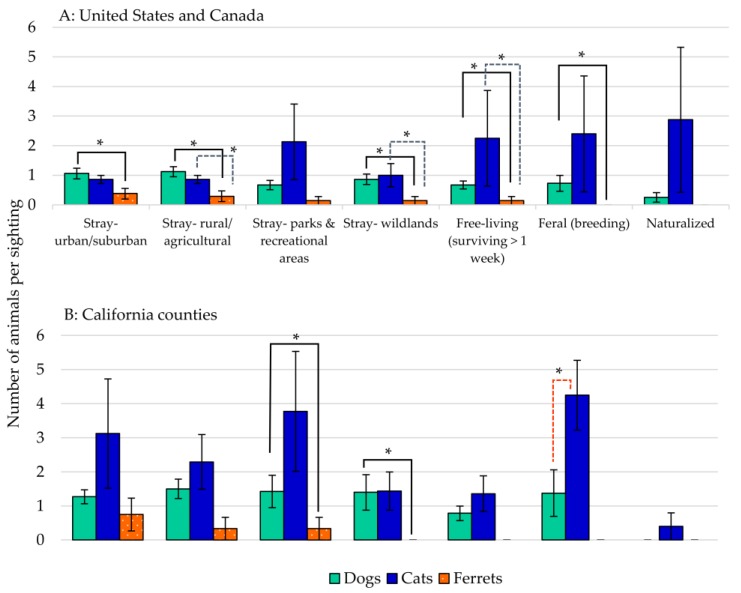
Average number of animals per sighting of (**A**) United States, Canada, and (**B**) California counties. Rating mean ± standard error of the mean (sem). Asterisk represents significant difference; * *p* < 0.05.

For “average numbers of unconfined animals” reported at each sighting (Figure 2, Table 1), dogs were most often sighted as single animals, whereas cats were seen in groups of three or more, with increasing numbers of cats observed as “free-living”, “feral (breeding)”, and “naturalized” animals. In “urban-suburban” and rural-agricultural settings, state and provincial agencies reported dogs and cats in equal numbers, whereas the California county agencies observed increased numbers of cats in these areas. For most other areas, cats were seen in greater numbers than dogs, and large numbers of feral cats were observed by all of the agencies. Except for occasional strays in areas of human activity, no California agency reported ferrets as “naturalized”, “feral (breeding)”, or “free-living”.

### 3.2. Sighting of Unconfined Animals—”Degree of Concern”

Respondents were asked to rank their “degree of concern” regarding the existence of unconfined dogs, cats, and ferrets. Ratings were quantified as follows: “definite concern” (−2), “some concern” (−1), “no concern” (0), “some benefit” (+1), or “definite benefit” (+2). For state and provincial agencies, cats and dogs engendered “some concern” to “definite concern” (Table 2). Feral cats were ranked the highest “degree of concern” (−1.83). Overall, unconfined cats ranked higher than dogs, with ferrets given the lowest level of concern (Overall Means: cats: −1.73; dogs: −1.47; ferrets: −0.56).

Ratings from the California county agencies were somewhat lower than those given by states and provinces (Overall Means: cats: −1.23; dogs: −1.21; ferrets: −0.43). The greatest “degree of concern” was from unconfined dogs in recreational (−1.74) and agricultural (−1.69) areas, and from unconfined stray cats in urban-suburban (−1.58) and in agricultural areas (−1.44), and in the feral state (−1.44). County agencies, which ranked ferrets lowest overall, most notably had the least concerns over “feral” (−0.36), or “naturalized” (−0.22) ferrets, which conformed to agencies’ lack of sightings or reports of ferrets existing in these states.

### 3.3. Impact: Existence of Incidents on Wildlife from Unconfined Animals and Degree of Concern

**Figure 3:**
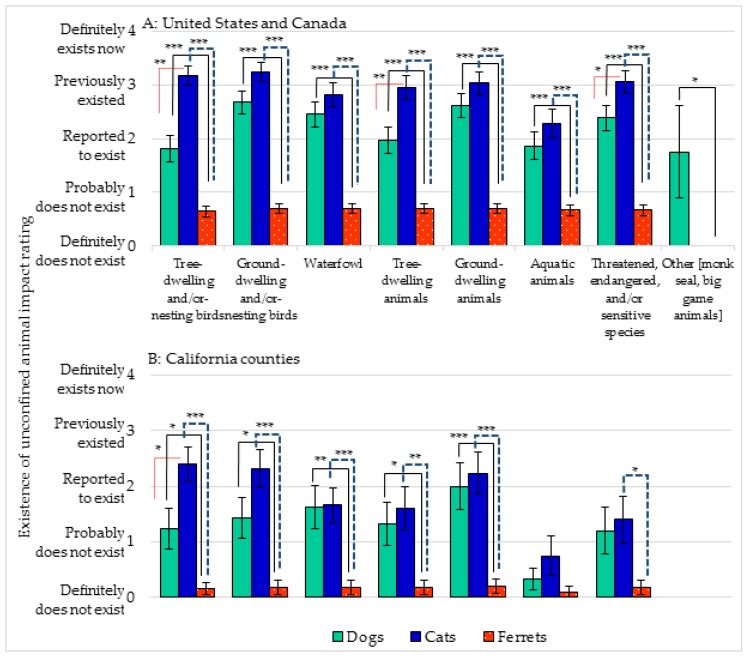
“Existence” of incidents (impact) on wildlife from unconfined dogs, cats, ferrets (**A**) United States, Canada, and (**B**) California counties. Rating mean ± standard error of the mean (sem). Asterisk represents significant difference; * *p* < 0.05, ** *p* < 0.01, and *** *p* < 0.001.

Respondents were also asked to identify the existence of incidents, or impact, from unconfined domestic carnivores on native wildlife (Figure 3; see also Supplementary Materials Table S1). State and provincial agencies ranked incidents from unconfined dogs and cats as “reported to exist” or “previously existed” regarding “tree-dwelling and/or nesting birds”, “ground-dwelling and/or nesting birds”, “waterfowl”, “tree-dwelling” animals, “ground-dwelling” animals”, “aquatic animals”, “threatened, endangered and/or sensitive species”, with Hawaii listing dogs as aggravating “monk” seals. With the exception of the “monk” seal (Hawaii) and “big game” animals, cats received the highest levels of incidents, representing the greatest negative impact on wildlife for all categories (cats: 2.53; dogs: 2.19). In contrast, ferrets ranged from “probably does not exist” to “definitely does not exist” (ferrets: 0.59). Results from the California county agencies followed a similar pattern, with ferret-related incidents ranked the lowest (cats: 1.55; dogs: 1.15; ferrets: 0.15).

State and provincial agencies ranked “degree of concern” highest for cats for their impact on “tree-dwelling” and “ground dwelling” birds, with the exception of Hawaii, which ranked dogs as a “definite concern” with respect to the “monk” seal (“other”) (Table 3). The California county agencies also ranked cats the highest with regard to the “degree of concern”, although they expressed at least “some concern” for all three species when unconfined with regard to “threatened, endangered and/or sensitive species”.

### 3.4. Impact: Existence of Incidents on Wildlife in Parks—Recreational Areas from Unconfined Animals and Degree of Concern

**Figure 4:**
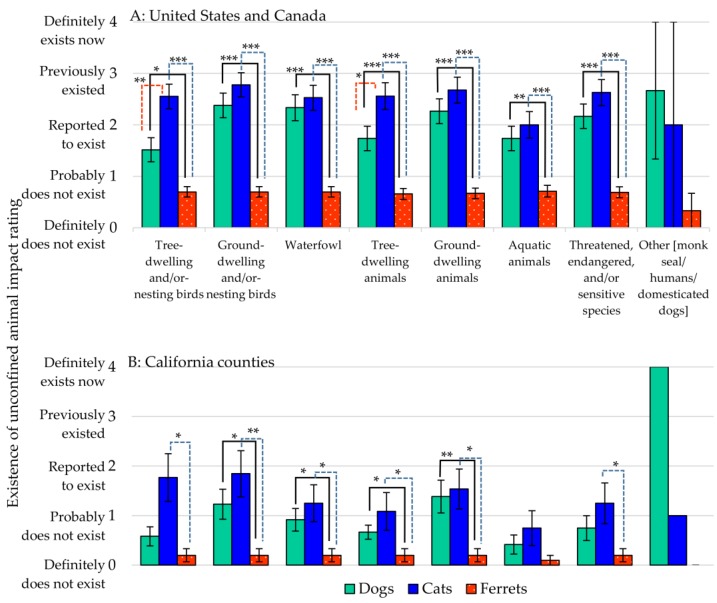
“Existence” of incidents (impact) on wildlife in parks and recreational areas from unconfined dogs, cats, ferrets (**A**) United States, Canada, and (**B**) California counties. Rating mean ± standard error of the mean (sem). Asterisk represents significant difference; * *p* < 0.05, ** *p* < 0.01, and *** *p* < 0.001.

Impact ratings on the wildlife in parks and recreational areas followed a pattern similar to the other environmental regions (Figure 4; see also Supplementary Materials Table S2). Cats featured more prominently than dogs in all of the wildlife categories for state and provincial agencies, as well as the California county agencies, with the exception of “other” animals. Dogs were listed as having a greater impact on the “monk” seal and “big game”. Interestingly, under “other”, the California counties noted that both cats, dogs (“on leashes”) and humans as having impact in recreational areas.

State and provincial agencies also ranked “degree of concern” highest for cats for their impact on “tree-dwelling” and “ground dwelling” birds, with the exception of Hawaii, which ranked dogs as a “definite concern” with respect to the “monk” seal (“other”). Similarly, the California county agencies ranked cats the highest with regard to “degree of concern”, although they expressed at least “some concern” for all three species when unconfined with regard to “threatened, endangered and/or sensitive species” (Table 4).

### 3.5. Classifications

Table 5 shows the terms used by agencies to classify dogs, cats, and ferrets. Overwhelmingly, dogs and cats were classified as “house-pet” or “companion” animal by both state and provincial agencies (>76%) and county agencies of California (90%), in contrast to ferrets (54% and 10%, respectively). There was much greater recognition of dogs and cats as “domestic” or “domesticated” (>80%), versus ferrets (states/provinces: 61%; California counties: 14%). Ferrets were frequently identified as “exotic” animals (states/provinces: 26%; California counties: 59%), whereas dogs and cats were not (0–3%). A minority of state and provincial agencies classified ferrets as “listed, restricted, not permitted” (7%) or “restricted, except under permit” (3%), whereas most, but not all, California county agencies classified ferrets as such (38% and 41%, respectively).

### 3.6. Actions Taken

Agencies were asked to list the actions undertaken when a free-roaming animal is found in their jurisdiction. Overall, agencies reported a wide-range of actions taken (Table 6). California county agencies reported that free-roaming dogs and cats were assumed to be lost pets (dogs: 67%, cats: 55%) that were trapped and then taken to shelters. At the state-provincial level, 40% of responders reported making “referrals to another agency”, which likely may have included animal control at a more local level. Despite reports of negative effects from free-roaming dogs and cats on wildlife and a high “degree of concern”, a small number of agencies reported that they would not take action, as it was “not considered to be important”. For free-roaming cats, 21% state/provincial agencies and 12% California county agencies reported that no action would be taken, as it was “desirable, but not feasible” to do so.

### 3.7. “Special Concerns”

“Special concerns” were provided by 52 (35-state/provincial; 17 California county) agencies. The majority of commenters at the state and provincial levels represented fish and wildlife agencies, and at the county level, animal control. Cats were mentioned most often (22–43%), with “feral” cats listed as a major concern based on their impact on wildlife and disease transmission, such as rabies, followed by dogs (13–25%), and specifically dogs “off-leash”. Only six (3-state/provincial and 3-county) agencies mentioned ferrets. Commenters from state/provincial agencies called out that no “feral” or “wild ferrets” existed within their jurisdiction, or that ferrets posed little or no concern. One California county agency expressed concerns that unconfined ferrets and cats could potentially cause harm to native animals; two counties commented that ferrets were “not legal” in the state.

## 4. Discussion

This is the first survey to compare the environmental impact of three non-native domestic carnivores. While other surveys have addressed the impact of free-roaming dogs, cats or ferrets, none has conducted a head-to-head comparison nor described the relative impact of these carnivores on the wildlife of North America [14,32,33]. A key advantage of this survey over those conducted by governmental bodies or well-known organizations is the anonymity of SeaSearch; which, holds no public position or political agenda that would influence the responder. Due to the uniformity of the respondents; who had similar roles and backgrounds in their respective agencies; as well as the relatively small number of nonresponders; concern regarding “non-response” bias was minimized [34,35]. Limitations to this study, however, include the inherent inaccuracies of retrospective reporting (“recall error”), intra-observer variation due to individual bias or experience, potential impact of local laws, regulation and practices, as well as a lack of documentation from respondents supporting their ratings [36,37].

The results from the current study showed remarkable consistency with prior reports. In this survey, the existence of feral cats and dogs was consistently reported, as well as their profound impact on local wildlife. Free-roaming cats were identified as having the greatest environmental impact. Under “special concerns” Hawaii stated “There are significantly more cats than there are dogs in free roaming populations”. One of the biologists estimates a 9:1 ratio. Iowa reported: “Our biggest concerns would be free-ranging or feral cats and their potential impact on wildlife”. New York voiced a concern of many of the states: “In the case of dogs and cats, these animals directly kill and injure countless numbers of small mammals and birds up to and including white-tailed deer…”. Quebec described its management efforts: “In the past years, we have had problems with racoon (sic) rabies. In an operation to control the disease, in southern Quebec in 2007, we have captured 10,000 racoons (sic) and 3500 domestic cats and only a few dogs…”.

Aside from an occasional “stray”, there were no reports of “feral (breeding)” ferrets; nor were there reports that of “free-living”, or “naturalized” ferrets anywhere in the continental United States or Canada, including California. More importantly, no jurisdiction reported ferrets impacting local (native) wildlife, including ground-nesting birds, or threatened, endangered and sensitive species.

Except for state requirements for licensing and routine immunization, most animal control activities are at the level of local government and the private sector. Cities, county parks and recreation, and housing and homeowners’ associations often impose “nuisance” and “leash” laws, limits on the number of allowable animals, and restrictions on ownership of certain dog breeds, such as pit bulls [38,39]. However, California, under a 1933 law, regulates ferrets as “wildlife”, prohibiting their sale or possession within the state [40]. Concern over the ferret’s invasive potential has been largely based on reports from island nations where deliberate introductions of species resulted in feral ferrets and other domestic species, which caused harm to the local ecosystems [30,31,40]. In its response to the current survey, neither the state nor its county agencies reported feral ferrets or environmental harm caused by ferrets, confirming the state’s own nation-wide survey [32]. The sum of the results calls into question the relevancy of insular experiences to a state that is contiguous with the North American continent.

Regardless, demands that “solid scientific evidence demonstrating no risk to our native wildlife and their habitats” regarding the ferret have continued [41], whereas most government agencies have taken little action to curtail free-roaming cats and dogs in the face of irrefutable evidence of profound negative environmental impact. Such regulatory bias may have less to do with objective data and more to do with human nature [42,43]. Public attitudes and opinions towards various animals have been shown to be affected by “the perceived attractiveness and usefulness of the species involved, indigenous or non-indigenous” [44]. The current study appears to support this conclusion. As “special concerns”, New York writes: “…If pesticides were the cause of the numbers of wild animals and birds injured or killed each year by domestic and feral house cats, the public would demand action to halt this destruction. However, when there is direct evidence of such wanton waste by domestic and feral house cats, the cat-lovers effectively threaten the elected officials from taking any actions to rid the environment of these introduced predators”.

Terminology used by agencies in describing these animals is also revealing. In the present study, dogs and cats are recognized as “house pets”—“companion” animals, and “domestic”—“domesticated” species (Table 3), whereas the ferret is considered “exotic”. “Exotic” means “foreign” and “not-native”, which makes the ferret no more “exotic” than the cat or dog (with the sole exception of the Alaskan malamute). The fact that the veterinary community refers to all small animals that are “not dogs or cats” as “exotic animals” undoubtedly contributes to the public’s view of these respective carnivores [45].

Moreover, in its current state code, California lists the ferret as a “detrimental” animal “not normally domesticated in this state”, whereas the Asian water buffalo (*Bubalus bubalis*), a relatively recent introduction to the state is considered a “welfare animal”, a mammal so “listed to prevent the depletion of wild populations and to provide for animal welfare” [46,47]. Such distinction reflects on societal values that go far beyond scientific principles to our cultural roots and perceptions and, on a more pragmatic level, to hardline economics: it is the water buffalo that produces a sought-after mozzarella cheese—not so for the ferret [48]. The labeling of the ferret as *not domestic* hence “wild” is not without consequence. Palmer argues that it changes the human-animal social contract, from one of caring and protection extended to domestic and companion animals, to a hands-off “laissez faire intuition”, that, as humans, intuitively we do not experience the same moral obligation towards an animal that by nature is “wild” [49].

The global policy initiatives to prevent and eradicate non-native species, which arose over concerns of “biological invasion”, are only recently undergoing re-examination [3,50,51]. Not only is there growing disagreement over what constitutes “harm”, arguably some species can be both “good” and “bad” for their new environment, “depending on the location and the perceptions of the observers” [13,50]. In 2006, the US National Invasive Species Council (NISC) cautioned: “Many alien species are non-invasive and support human livelihoods or a preferred quality of life” [44]. Under certain conditions, even native organisms have been shown to exhibit “invasiveness” within their own natural habitats [43]. More importantly, introduction of nonnative species has in some instances resulted in an increase in overall biodiversity [52].

NISC provides additional cautions: “Because invasive species management is difficult and often very expensive, (the) worst offenders are the most obvious and best targets for policy attention and management” [53]. Based on current evidence for the North American continent, the ferret is a low probability target, whereas unconfined dogs and cats, particularly in the feral state, should rank high among the list of management priorities. The true target, however, may be management of public attitudes and perceptions regarding these and other non-native species.

## 5. Conclusions

This survey confirms the negative impact from free-roaming cats and dogs on native North American wildlife, while demonstrating the absence of any discernible impact from the ferret. Based on evidence of substantial environmental impact and harm, unconfined cats and the dogs meet the definition for “invasive” species, whereas the ferret does not on this continent. Results from this study display the differences among three carnivore species and their relative impact on the North American ecosystem. Public attitudes and perceptions regarding these species, however, may play a role in agency control efforts and management priorities.

## Figures and Tables

**Table 1:** Number of animals per sighting.

Animal Sightings	Dogs	Cats	Ferrets	*p*-Value
	Mean ± sem	Mean ± sem	Mean ± sem	
United States and Canada				
Stray—urban/suburban	1.06 ± 0.18	0.86 ± 0.14	0.38 ± 0.18	^ac^ ns
				^b^ *p* < 0.05
Stray—rural/agricultural	1.12 ± 0.17	0.86 ± 0.14	0.29 ± 0.18	^a^ ns
				^bc^ *p* < 0.05
Stray—parks & recreational areas	0.67 ± 0.16	2.13 ± 1.28	0.14 ± 0.14	^abc^ ns
Stray—wildlands	0.86 ± 0.18	1.00 ± 0.39	0.14 ± 0.14	^a^ ns
				^bc^ *p* < 0.05
Free-living (surviving > 1 week)	0.67 ± 0.14	2.25 ± 1.62	0.14 ± 0.14	^a^ ns
				^bc^ *p* < 0.05
Feral (breeding)	0.73 ± 0.27	2.40 ± 1.96	0.00 ± 0.00	^ac^ ns
				^b^ *p* < 0.05
Naturalized	0.25 ± 0.16	2.88 ± 0.2.45	0.00 ± 0.00	^abc^ ns
California Counties				
Stray Animals—urban/suburban	1.27 ± 0.20	3.13 ± 1.60	0.75 ± 0.48	^abc^ ns
Stray Animals—rural/agricultural	1.50 ± 0.29	2.29 ± 0.80	0.33 ± 0.33	^abc^ ns
Stray animals—parks & recreational areas	1.42 ± 0.47	3.77 ± 1.76	0.33 ± 0.33	^ac^ ns
				^b^ *p* < 0.05
Stray Animals—wildlands	1.40 ± 0.52	1.44 ± 0.56	0.00 ± 0.00	^ac^ ns
				^b^ *p* < 0.05
Free-living animals (surviving > 1 week)	0.79 ± 0.21	1.36 ± 0.52	0.00 ± 0.00	^abc^ ns
Feral (Breeding) Animals	1.38 ± 0.68	4.25 ± 1.03	0.00 ± 0.00	^ac^ *p* < 0.05
				^b^ ns
Naturalized animals	0.00 ± 0.00	0.40 ± 0.40	0.00 ± 0.00	^ac^ ns

Statistical comparisons: ^a^ dogs vs.cats, ^b^ ferrets vs. dogs, ^c^ ferrets vs. cats; sem: standard error of the mean; ns: nonsignificant.

**Table 2:** “Degree of concern” regarding sightings of unconfined dogs, cats, ferrets.

Animal Sightings	Dogs		Cats		Ferrets		*p*-Value
	Mean ± sem	¹ Rating Range	Mean ± sem	Rating Range	Mean ± sem	Rating Range	
United States, DC and Canada							
Stray-urban/suburban	−1.58 ± 0.09	(−2)–0	−1.76 ± 0.08	(−2)–0	−0.50 ± 0.12	(−2)–0	ᵃ ns
							^bc^ *p* < 0.001
Stray-rural/ agricultural	−1.61 ± 0.09	(−2)–0	−1.72 ± 0.09	(−2)–1	−0.54 ± 0.14	(−2)–1	ᵃ ns
							^bc^ *p* < 0.001
Stray-parks & recreational areas	−1.57 ± 0.08	(−2)–0	−1.70 ± 0.09	(−2)–1	−0.56 ± 0.13	(−2)–0	ᵃ ns
							^bc^ *p* < 0.001
Stray-wildlands	−1.49 ± 0.10	(−2)–0	−1.70 ± 0.07	(−2)–(−1)	−0.58 ± 0.14	(−2)–0	ᵃ ns
							^bc^ *p* < 0.001
Free-living (surviving > 1 week)	−1.50 ± 0.10	(−2)–0	−1.76 ± 0.08	(−2)–0	−0.54 ± 0.13	(−2)–0	ᵃ ns
							^bc^ *p* < 0.001
Feral (Breeding)	−1.53 ± 0.10	(−2)–0	−1.83 ± 0.06	(−2)–(−1)	−0.54 ± 0.13	(−2)–0	ᵃ *p* < 0.05
							^bc^ *p* < 0.001
Naturalized	−1.03 ± 0.18	(−2)–2	−1.61 ± 0.13	(−2)–0	−0.65 ± 0.15	(−2)–0	ᵃ *p* < 0.05
							^b^ ns
							^c^ *p* < 0.001
Overall Means	−1.47		−1.73		−0.56		
California Counties							
Stray-urban/suburban	−1.00 ± 0.19	(−2)–0	−1.58 ± 0.16	(−2)–1	−0.47 ± 0.19	(−2)–0	ᵃ ns
							^bc^ *p* < 0.001
Stray-rural/agricultural	−1.74 ± 0.11	(−2)–0	−1.44 ± 0.20	(−2)–2	−0.47 ± 0.19	(−2)–0	ᵃ ns
							^bc^ *p* < 0.001
Stray-parks & recreational areas	−1.69 ± 0.13	(−2)–0	−1.36 ± 0.16	(−2)–1	−0.50 ± 0.20	(−2)–0	ᵃ ns
							^bc^ *p* < 0.001
							^c^ *p* < 0.01
Stray-wildlands	−1.28 ± 0.16	(−2)–0	–1.08 ± 0.19	(−2)–1	−0.46 ± 0.22	(−2)–0	ᵃ ns
							ᵇ *p* < 0.01
							^c^ *p* < 0.05
Free-living (surviving > 1 week)	−1.16 ± 0.18	(−2)–0	−1.19 ± 0.20	(−2)–1	−0.50 ± 0.23	(−2)–0	ᵃ ns
							^bc^ *p* < 0.05
Feral (Breeding)	−1.29 ± 0.18	(−2)–0	−1.44 ± 0.20	(−2)–1	−0.36 ± 0.20	(−2)–0	ᵃ ns
							^bc^ *p* < 0.01
Naturalized	−0.33 ± 0.26	(−2)–1	−0.50 ± 0.25	(−2)–1	−0.22 ± 0.22	(−2)–0	^abc^ ns
Overall Means	−1.21		−1.23		−0.43		

¹ Rating scale: “definite concern”: −2, “some concern”: −1, “no concern”: 0, “some benefit”: +1, “definite benefit”: +2; Statistical comparisons: ᵃ dogs vs. cats, ᵇ ferrets vs. dogs, ^c^ ferrets vs. cats; sem: standard error of the mean; ns: nonsignificant.

**Table 3:** “Degree of concern” regarding the effects (impact) of unconfined animal on wildlife.

Effects on Wildlife	Dogs		Cats		Ferrets		*p*-Value
	Mean ± sem	^1^ Rating Range	Mean ± sem	Rating Range	Mean ± sem	Rating Range	
United States, DC and Canada							
Tree-Dwelling and/or–nesting birds	−0.68 ± 0.13	(−2)–0	−1.78 ± 0.07	(−2)–(−1)	−0.33 ± 0.14	(−2)–0	^ac^ *p* < 0.001
							ᵇ ns
Ground-dwelling and/or–nesting birds	−1.43 ± 0.10	(−2)–0	−1.90 ± 0.05	(−2)–(−1)	−0.56 ± 0.18	(−2)–0	^abc^ *p* < 0.001
Waterfowl	−1.09 ± 0.11	(−2)–0	−1.41 ± 0.11	(−2)–0	−0.33 ± 0.11	(−1)–0	ᵃ *p* < 0.05
							ᵇ^c^ *p* < 0.001
Tree-dwelling animals	−0.75 ± 0.11	(−2)–0	−1.28 ± 0.14	(−2)–0	−0.44 ± 0.15	(−2)–0	ᵃ *p* < 0.01
							ᵇ ns
							^c^ *p* < 0.001
Ground-dwelling animals	−1.36 ± 0.10	(−2)–0	−1.62 ± 0.09	(−2)–0	−0.56 ± 0.18	(−2)–0	ᵃ ns
							^bc^ *p* < 0.001
Aquatic animals	−0.54 ± 0.11	(−2)–0	−0.63 ± 0.12	(−2)–0	−0.22 ± 0.10	(−1)–0	ᵃᵇ ns
							^c^ *p* < 0.05
Threatened, Endangered, and/or Sensitive species	−1.13 ± 0.13	(−2)–0	−1.63 ± 0.13	(−2)–0	−0.63 ± 0.21	(−2)–0	ᵃ *p* < 0.01
							ᵇ *p* < 0.05
							^c^ *p* < 0.001
Other (Monk Seal, Big Game Animals)	−1.33 ± 0.33	(−2)–(−1)	0	0	0	0	ᵇ ns
Overall Means	−1.04		−1.28		−0.38		
California Counties							
Tree-Dwelling and/or—nesting birds	−0.11 ± 0.35	(−2)–2	−1.64	(−2)–(−1)	−0.33	(−1)–0	ᵃ *p* < 0.001
							ᵇ ns
							^c^ *p* < 0.01
Ground-dwelling and/or—nesting birds	−1.09 ± 0.28	(−2)–0	−1.46 ± 0.18	(−2)–0	−0.33 ± 0.33	(−1)–0	ᵃᵇ ns
							^c^ *p* < 0.05
Waterfowl	−1.09 ± 0.28	(−2)–0	−1.30 ± 0.26	(−2)–0	−0.33 ± 0.33	(−1)–0	^abc^ ns
Tree-dwelling animals	−0.78 ± 0.32	(−2)–0	−1.25 ± 0.37	(−2)–0	−0.33 ± 0.33	(−1)–0	^abc^ ns
Ground-dwelling animals	−1.31 ± 0.21	(−2)–0	−1.40 ± 0.22	(−2)–0	−0.33 ± 0.33	(−1)–0	^abc^ns
Aquatic animals	−0.50 ± 0.34	(−2)–0	−0.86 ± 0.34	(−2)–0	−0.33 ± 0.33	(−1)–0	^abc^ ns
Threatened, Endangered, and/or Sensitive species	−1.30 ± 0.30	(−2)–0	−1.44 ± 0.29	(−2)–0	−0.33 ± 0.33	(−1)–0	^abc^ ns
Other (Monk Seal, Big Game Animals)	0	0	0	0	0	0	-
Overall Means	−0.77		−1.17		−0.29		

¹ Rating scale: “definite concern”: −2, “some concern”: −1, “no concern”: 0, “some benefit”: +1, “definite benefit”: +2; Statistical comparisons: ᵃ dogs vs. cats, ᵇ ferrets vs. dogs, ^c^ ferrets vs. cats; sem: standard error of the mean; ns: nonsignificant.

**Table 4:** “Degree of concern” regarding the effects (impact) of unconfined animal on wildlife in parks and recreational areas.

Effects on Parks/Recreational Areas	Dogs		Cats		Ferrets		*p*-Value
	Mean ± sem	^1^ Rating Range	Mean ± sem	Rating Range	Mean ± sem	Rating Range	
United States, DC and Canada							
Tree-Dwelling and/or–nesting birds	−0.54 ± 0.12	(−2)–0	−1.35 ± 0.16	(−2)–0	−0.64 ± 0.17	(−2)–0	ᵃ *p* < 0.001
							ᵇ ns
							^c^ *p* < 0.01
Ground-dwelling and/or–nesting birds	−1.25 ± 0.14	(−2)–0	−1.57 ± 0.13	(−2)–0	−0.36 ± 0.20	(−2)–0	ᵃ ns
							ᵇ^c^ *p* < 0.001
Waterfowl	−0.96 ± 0.14	(−2)–0	−1.15 ± 0.15	(−2)–0	−0.07 ± 0.07	(−1)–0	ᵃ ns
							^bc^ *p* < 0.001
Tree-dwelling animals	−0.43 ± 0.12	(−2)–0	−1.20 ± 0.15	(−2)–0	−0.23 ± 0.17	(−2)–0	^ac^ *p* < 0.001
							ᵇ ns
Ground-dwelling animals	−1.19 ± 0.12	(−2)–0	−1.42 ± 0.14	(−2)–0	−0.29 ± 0.16	(−2)–0	ᵃ ns
							^bc^ *p* < 0.001
Aquatic animals	−0.45 ± 0.14	(−2)–0	−0.45 ± 0.14	(−2)–0	−0.13 ± 0.09	(−1)–0	^abc^ ns
Threatened, Endangered, and/or Sensitive species	−1.12 ± 0.17	(−2)–0	−1.44 ± 0.16	(−2)–0	−0.43 ± 0.20	(−2)–0	ᵃ ns
							ᵇ *p* < 0.05
							^c^ *p* < 0.01
Other (monk seal/humans/domesticated dogs)	−2.00	−2	-	-	-	-	-
Overall Mean:	−0.99		−1.23		−0.31		
California Counties							
Tree-Dwelling and/or- nesting birds	−0.33 ± 0.33	(−2)–0	−1.11 ± 0.31	(−2)–0	−0.67 ± 0.67	(−2)–0	^abc^ ns
Ground-dwelling and/or- nesting birds	−0.89 ± 0.31	(−2)–0	−1.33 ± 0.29	(−2)–0	−0.67 ± 0.67	(−2)–0	^abc^ ns
Waterfowl	−0.71 ± 0.36	(−2)–0	−1.17 ± 0.40	(−2)–0	−0.67 ± 0.67	(−2)–0	^abc^ ns
Tree-dwelling animals	−0.33 ± 0.33	(−2)–0	−1.00 ± 0.45	(−2)–0	−0.67 ± 0.67	(−2)–0	^abc^ ns
Ground-dwelling animals	−0.89 ± 0.26	(−2)–0	−1.13 ± 0.30	(−2)–0	−0.67 ± 0.67	(−2)–0	^abc^ ns
Aquatic animals	−0.50 ± 0.34	(−2)–0	−0.71 ± 0.36	(−2)–0	−0.50 ± 0.50	(−2)–0	^abc^ ns
Threatened, Endangered, and/or Sensitive species	−1.00 ± 0.38	(−2)–0	−1.29 ± 0.36	(−2)–0	−0.67 ± 0.67	(−2)–0	^abc^ ns
Other (monk seal/humans/domesticated dogs)	-	-	-	-	-	-	-
Overall Mean:	−0.67		−1.11		−0.64		

¹ Rating scale: “definite concern”: −2, “some concern”: −1, “no concern”: 0, “some benefit”: +1, “definite benefit”: +2; Statistical comparisons: ᵃ dogs vs. cats, ᵇ ferrets vs. dogs, ^c^ ferrets vs. cats; sem: standard error of the mean; ns: nonsignificant.

**Table 5:** Terms used by agencies to classify dogs, cats, ferrets.

Classification	Dogs	Cats	Ferrets
United States, DC and Canada			
“House-pet” or “Companion”	56 (78%)	55 (76%)	39 (54%)
“Domestic” or “Domesticated”	59 (82%)	58 (81%)	44 (61%)
“Exotic animal”	1 (1%)	2 (3%)	19 (26%)
“Non-game”	9 (13%)	9 (13%)	10 (14%)
“Fur-bearing”	0	1 (1%)	8 (11%)
“Laboratory” or “Research”	13 (18%)	13 (18%)	9 (13%)
“Wild” or Wildlife”	2 (3%)	2 (3%)	7 (10%)
“Unlisted” or “Unrestricted” or “Permitted”	10 (14%)	9 (13%)	14 (19%)
“Listed” or “Restricted” or “Not permitted”	3 (4%)	2 (3%)	2 (3%)
“Restricted, except under a permit”	0	0	5 (7%)
Other (Please specify in Comments)	1 (1%)	2 (3%)	2 (3%)
No Classification	9 (13%)	11 (15%)	12 (17%)
California Counties			
“House- pet” or “Companion”	26 (90%)	26 (90%)	3 (10%)
“Domestic” or “Domesticated”	26 (90%)	25 (86%)	4 (14%)
“Exotic animal”	0	1 (3%)	17 (59%)
“Non-game”	12 (41%)	11 (38%)	9 (31%)
“Fur-bearing”	2 (7%)	2 (7%)	8 (28%)
“Laboratory” or “Research”	8 (28%)	7 (24%)	3 (10%)
“Wild” or Wildlife”	2 (7%)	1 (3%)	8 (28%)
“Unlisted” or “Unrestricted” or “Permitted”	6 (21%)	6 (21%)	2 (7%)
“Listed” or “Restricted” or “Not permitted”	1 (3%)	1 (3%)	11 (38%)
“Restricted, except under a permit”	1 (3%)	0	12 (41%)
Other (Please specify in Comments)	0	0	1 (3%)
No Classification	3 (10%)	3 (10%)	2 (7%)

**Table 6:** Actions taken by agencies for unconfined dogs, cats, ferrets.

Action Taken	Dogs	Cats	Ferrets
United States, DC, and Canada			
None: not considered to be important	2 (3%)	3 (4%)	8 (11%)
None: desirable, but not feasible	12 (17%)	15 (21%)	9 (13%)
Live trap/take to shelter (assumed lost pet)	17 (24%)	15 (21%)	8 (11%)
Live trap/euthanize	8 (11%)	10 (14%)	7 (10%)
Live trap/transport (specify destination in Comments)	7 (10%)	9 (13%)	4 (6%)
Take by any means	10 (14%)	5 (7%)	4 (6%)
Refer to another agency (specify in Comments)	27 (38%)	24 (34%)	19 (27%)
Attempt eradication	3 (4%)	4 (6%)	4 (6%)
Unknown	3 (4%)	4 (6%)	8 (11%)
California Counties			
None: not considered to be important	1 (3%)	3 (9%)	2 (6%)
None: desirable, but not feasible	0	4 (12%)	0
Live trap/take to shelter (assumed lost pet)	22 (67%)	18 (55%)	6 (18%)
Live trap/euthanize	7 (21%)	7 (21%)	5 (15%)
Live trap/transport (specify destination in Comments)	4 (12%)	3 (9%)	2 (6%)
Take by any means	5 (15%)	2 (6%)	4 (12%)
Refer to another agency (specify in Comments)	4 (12%)	4 (12%)	13 (40%)
Attempt eradication	2 (6%)	1 (3%)	2 (6%)
Unknown	0	0	1 (3%)

## Supplementary Materials

The following are available online at http://www.mdpi.com/2076-2615/7/10/78/s1: the supplementary questionnaire; Table S1: Existence of incidents of unconfined dogs, cats, ferrets: impact on wildlife, Table S2: Existence of incidents of unconfined dogs, cats, ferrets: impact on wildlife in parts-recreational areas.
