# Supplementary material for: Environmental Impact and Relative Invasiveness of Free-Roaming Domestic Carnivores—a North American Survey of Governmental Agencies
Source: Animals (Basel). 2017 Oct 14;7(10):78. doi: 10.3390/ani7100078 (PMC5664037; doi:10.3390/ani7100078)
Supplement: Supplementary file 1 [file animals-07-00078-s001.pdf]

The following Supplemental Tables represent the data upon which Figures 3 and 4, respectively, are based.

Table S1: Existence of incidents of unconfined dogs, cats, ferrets: impact on wildlife

Effects on Wildlife	Dogs		Cats		Ferrets		p-value
	Mean ± sem	Rating Range	Mean ± sem	Rating Range	Mean ± sem	Rating Range	
**United States, DC and Canada**							
Tree-dwelling and/or- nesting birds	1.82 ±0.24	0-4	3.16 ±0.18	0-4	0.65 ±0.10	0-2	[abc] $p<0.001$
Ground-dwelling and/or- nesting birds	2.68 ±0.21	0-4	3.23 ±0.18	0-4	0.69 ±0.10	0-2	[a] ns<br>[bc] $p<0.001$
Waterfowl	2.45 ±0.24	0-4	2.82 ±0.22	0-4	0.69 ±0.10	0-2	[a] ns<br>[bc] $p<0.001$
Tree-dwelling animals	1.97 ±0.25	0-4	2.95 ±0.23	0-4	0.69 ±0.10	0-2	[a] $p<0.01$<br>[bc] $p<0.001$
Ground-dwelling animals	2.62 ±0.22	0-4	3.03 ±0.21	0-4	0.69 ±0.10	0-2	[a] ns<br>[bc] $p<0.001$
Aquatic animals	1.86 ±0.26	0-4	2.27 ±0.27	0-4	0.67 ±0.10	0-2	[a] ns<br>[bc] $p<0.001$
Threatened, endangered, and/or sensitive species	2.38 ±0.23	0-4	3.05 ±0.22	0-4	0.67 ±0.10	0-2	[a] $p<0.05$<br>[bc] $p<0.001$
Other [monk seal, big game animals]	1.75 ±0.85	0-4	0.00	0	0.00	0	-
Overall Mean:	**2.19**		**2.56**		**0.59**		
**California counties**							
Tree-dwelling and/or- nesting birds	1.24 ±0.36	0-4	2.40 ±0.30	0-4	0.17 ±0.11	0-1	[ab] $p<0.05$<br>[c] $p<0.001$
Ground-dwelling and/or- nesting birds	1.44 ±0.36	0-4	2.32 ±0.33	0-4	0.18 ±0.12	0-1	[a] ns<br>[b] $p<0.05$<br>[c] $p<0.001$
Waterfowl	1.63 ±0.40	0-4	1.67 ±0.21	0-4	0.18 ±0.12	0-1	[a] ns<br>[b] $p<0.01$<br>[c] $p<0.001$
Tree-dwelling animals	1.33 ±0.39	0-4	1.60 ±0.39	0-4	0.18 ±0.12	0-1	[a] ns<br>[b] $p<0.05$<br>[c] $p<0.01$
Ground-dwelling animals	2.00 ±0.42	0-4	2.24 ±0.38	0-4	0.20 ±0.13	0-1	[a] ns<br>[bc] $p<0.001$
Aquatic animals	0.33 ±0.19	0-2	0.75 ±0.35	0-4	0.10 ±0.10	0-1	[abc] ns
Threatened, endangered, and/or sensitive species	1.20 ±0.42	0-4	1.40 ±0.41	0-4	0.18 ±0.12	0-1	[ab] ns<br>[c] $p<0.05$
Other [monk seal, big game animals]	0.00	0	0.00	0	0.00	0	-
Overall Mean:	**1.15**		**1.55**		**0.15**		

Rating scale: “definite concern”: -2, “some concern”: -1, “no concern”: 0, “some benefit”: +1, “definite benefit”: +2;); Statistical comparisons: [a] dogs vs. cats, [b] ferrets vs. dogs, [c] ferrets vs. cats; sem: standard error of the mean; ns: nonsignificant.

Table S2: Existence of incidents of unconfined dogs, cats, ferrets: impact on wildlife in parts and recreational areas

Effects on Parks/Beaches	Dogs		Cats		Ferrets		p-value
	Mean ± sem	Rating Range	Mean ± sem	Rating Range	Mean ± sem	Rating Range	
**United States, DC and Canada**							
Tree-dwelling and/or- nesting birds	1.51 ±0.23	0-4	2.56 ±0.24	0-4	0.70 ±0.10	0-2	[a] $p<0.01$ [b] $p<0.05$ [c] $p<0.001$
Ground-dwelling and/or- nesting birds	2.38 ±0.24	0-4	2.78 ±0.24	0-4	0.70 ±0.10	0-2	[a] ns [bc] $p<0.001$
Waterfowl	2.33 ±0.25	0-4	2.53 ±0.24	0-4	0.70 ±0.10	0-2	[a] ns [bc] $p<0.001$
Tree-dwelling animals	1.74 ±0.24	0-4	2.56 ±0.26	0-4	0.66 ±0.11	0-2	[a] $p<0.05$ [bc] $p<0.001$
Ground-dwelling animals	2.26 ±0.24	0-4	2.68 ±0.25	0-4	0.67 ±0.10	0-2	[a] ns [bc] $p<0.001$
Aquatic animals	1.74 ±0.24	0-4	2.00 ±0.26	0-4	0.71 ±0.12	0-2	[a] ns [bc] $p<0.001$
Threatened, endangered, and/or sensitive species	2.17 ±0.24	0-4	2.63 ±0.25	0-4	0.69 ±0.10	0-2	[a] ns [bc] $p<0.001$
Other [monk seal/ humans/ domesticated dogs]	2.67 ±1.33	0-4	2.00 ±2.00	0-4	0.33 ±0.33	0-2	[abc] ns
Overall Mean:	**2.10**		**2.47**		**0.64**		
**California counties**							
Tree-dwelling and/or- nesting birds	0.58 ±0.19	0-2	1.77 ±0.48	0-4	0.20 ±0.13	0-1	[ac] $p<0.05$ [b] ns
Ground-dwelling and/or- nesting birds	1.23 ±0.30	0-3	1.85 ±0.46	0-4	0.20 ±0.13	0-1	[a] ns [bc] $p<0.01$
Waterfowl	0.92 ±0.23	2	1.25 ±0.37	0-4	0.20 ±0.13	0-1	[a] ns [bc] $p<0.05$
Tree-dwelling animals	0.67 ±0.14	0-1	1.08 ±0.38	0-4	0.20 ±0.13	0-1	[a] ns [bc] $p<0.05$
Ground-dwelling animals	1.38 ±0.33	0-4	1.54 ±0.40	0-4	0.20 ±0.13	0-1	[a] ns [bc] $p<0.01$
Aquatic animals	0.42 ±0.19	0-2	0.75 ±0.35	0-4	0.10 ±0.10	0-1	[abc] ns
Threatened, endangered, and/or sensitive species	0.75 ±0.25	0-2	1.25 ±0.41	0-4	0.20 ±0.13	0-1	[ab] ns [c] $p<0.05$
Other [monk seal/ humans/ domesticated dogs]	4.00	4	1.00	1	0.00	0	-
Overall Mean:	**1.24**		**1.31**		**0.16**		

Rating scale: “definite concern”: -2, “some concern”: -1, “no concern”: 0, “some benefit”: +1, “definite benefit”: +2;); Statistical comparisons: [a] dogs vs. cats, [b] ferrets vs. dogs, [c] ferrets vs. cats; sem: standard error of the mean; ns: nonsignificant.

SeaSearch Biological Surveys

DOMESTIC CARNIVORE SURVEY

AGENCY INFORMATION

Federal [ ] State/Province [ ] Other [ ] ______________________________

Agency:____

____

Division/Unit:____

____

RESPONDER'S NAME:

First:____

Middle Int.______

Last:____

Title:____

MAILING ADDRESS:

____

____

____

____

TELEPHONE (w/ Country-City/Area Codes AND Extension):

____

FACSIMILE:

____

E-MAIL:

____

WEBSITE (if applicable):

____

THROUGH OUT: ATTACH ADDITIONAL PAGES, AS NEEDED.

SeaSearch Biological Surveys

DOMESTIC CARNIVORE SURVEY

CLASSIFICATION

Which terms or classification(s) apply to EACH of these animals? Check ALL that apply:

NAME(S)		ANIMAL		
		**Dogs**	**Cats**	**Ferrets**
I	“House-Pet” or “Companion”			
II	“Domestic” or “Domesticated”			
III	“Exotic animal”			
IV	“Non-game”			
V	“Fur-bearing”			
VI	“Laboratory” or “Research”			
VII	“Wild” or “Wildlife”			
VII	“Listed” or ”Unrestricted” or “Permitted”			
IX	“Unlisted ” or ”Restricted” or “Not permitted”			
X	“Restricted, except under a permit”			
XI*	Other* [Please provide term]			
XII	[None]			

*Comments: Please provide clarification and details, as necessary.

____

____

____

____

____

____

____

____

____

ATTACH ADDITIONAL PAGES, AS NEEDED.

SeaSearch Biological Surveys

DOMESTIC CARNIVORE SURVEY

ANIMAL SIGHTINGS **NOT APPLICABLE [ ]**

Please identify SIGHTINGS of UNCONFINED ANIMALS in your jurisdiction.

RATING: ***"Animals have been seen…"***

0 Never
1 Historically [no in past 10 years]
2 Rarely [< 1 time per year]
3 Commonly [>1 time per year]
4 Frequently [> 5 times per month]
5 Unknown

Where appropriate, please add <u>AVERAGE NUMBER</u> OF ANIMALS PER SIGHTING

SIGHTINGS		ANIMAL					
		Dogs		Cats		Ferrets	
		Rating	# of animals	Rating	# of animals	Rating	# of animals
I	Stray animals – urban/suburban						
II	Stray animals – rural/agricultural						
III	Stray animals – parks and recreational areas						
IV	Stray animals – wild lands						
V	Free-living animals (surviving > 1 week)						
VI	Feral (breeding) animals						
VII	Naturalized animals						

Comments: Please provide clarification and details, as necessary.

____

____

____

____

ATTACH ADDITIONAL PAGES, AS NEEDED.

SeaSearch Biological Surveys

DOMESTIC CARNIVORE SURVEY

SIGHTINGS OF UNCONFINED ANIMAL POPULATIONS

Please identify SIGHTINGS of unconfined animals in your jurisdiction.

RATING: ***"Unconfined animals…."***

**0**	**Definitely do not exist**	**2**	**Reported to exist**
**1**	**Probably do not exist**	**3**	**Previously existed, do not exist now**
		**4**	**Definitely exists now**

For RATINGS 2 through 4, please also rate EFFECTS:

**A**	**No concern**	**D**	**Some benefit**	**F**	**Don't Know**
**B**	**Some concern**	**E**	**Definitely a benefit**	**G**	**No opinion**

SIGHTINGS		ANIMAL					
		Dogs		Cats		Ferrets	
		Rating	Effect	Rating	Effect	Rating	Effect
I	Stray animals – urban/suburban areas						
II	Stray animals – rural/agricultural						
III	Stray animals – parks and recreational areas						
IV	Stray animals – wild lands						
V	Free-living animals (surviving > 1 week)						
VI	Feral (breeding) animals						
VII	Naturalized animals						

Comments: Please provide documentation, if possible.

____

____

____

SeaSearch Biological Surveys

DOMESTIC CARNIVORE SURVEY

EFFECTS ON WILDLIFE **NOT APPLICABLE [ ]**

Please identify INCIDENTS of unconfined animals in your jurisdiction. ***Please check NOT APPLICABLE if this is not pertinent to your Agency or Jurisdiction.***

RATING: ***"Unconfined animals…."***

**0**	**Definitely do not exist**	**2**	**Reported to exist**
**1**	**Probably do not exist**	**3**	**Previously existed, do not exist now**
		**4**	**Definitely exists now**

For RATINGS 2 through 4, please also rate EFFECTS:

**A**	**No concern**	**D**	**Some benefit**	**F**	**Don't Know**
**B**	**Some concern**	**E**	**Definitely a benefit**	**G**	**No opinion**
**C**	**Definitely a concern**				

**Animal Group**		**ANIMAL**					
		**Dogs**		**Cats**		**Ferrets**	
		Rating	Effect	Rating	Effect	Rating	Effect
I	Tree-dwelling/and/or nesting birds						
II	Ground-dwelling and/or nesting birds						
III	Water fowl						
IV	Tree-dwelling animals						
V	Ground-dwelling animals						
VI	Aquatic animals						
VII	Threatened, Endangered, and/or Sensitive species						
VIII	Other: [list/rate]						
IX							
X							

Comments: Please provide documentation, if possible.

____

____

____

____

____

ATTACH ADDITIONAL PAGES, AS NEEDED.

SeaSearch Biological Surveys

DOMESTIC CARNIVORE SURVEY

EFFECTS ON PARKS/BEACHES **NOT APPLICABLE [ ]**

Please identify INCIDENTS of unconfined animals in your jurisdiction. ***Please check NOT APPLICABLE if this is not pertinent to your Agency or Jurisdiction.***

RATING: ***"Unconfined animals…."***

**0**	**Definitely do not exist**	**2**	**Reported to exist**
**1**	**Probably do not exist**	**3**	**Previously existed, doe not exist now**
		**4**	**Definitely exists now**

For RATINGS 2 through 4, please also rate EFFECTS:

**A**	**No concern**	**D**	**Some benefit**	**F**	**Don't Know**
**B**	**Some concern**	**E**	**Definitely a benefit**	**G**	**No opinion**
**C**	**Definitely a concern**				

**Animal Group**		**ANIMAL**					
		**Dogs**		**Cats**		**Ferrets**	
		Rating	Effect	Rating	Effect	Rating	Effect
I	Tree-dwelling/and/or nesting birds						
II	Ground-dwelling and/or nesting birds						
III	Water fowl						
IV	Tree-dwelling animals						
V	Ground-dwelling animals						
VI	Aquatic animals						
VII	Threatened, Endangered, and/or Sensitive species						
VIII	Other: [list/rate]						
IX							
X							

Comments: Please provide documentation, if possible.

____

____

____

____

____

____

ATTACH ADDITIONAL PAGES, AS NEEDED.

SeaSearch Biological Surveys

DOMESTIC CARNIVORE SURVEY

The following questions concern specific INCIDENTS AND EFFECTS. Please check NOT APPLICABLE if this is not pertinent to your Agency or Jurisdiction.

EFFECTS ON AGRICULTURE **NOT APPLICABLE [ ]**

Please identify INCIDENTS of unconfined animals in your jurisdiction.

RATING: ***"Unconfined animals…."***

**0**	**Definitely do not exist**	**2**	**Reported to exist**
**1**	**Probably do not exist**	**3**	**Previously existed, do not exist now**
		**4**	**Definitely exists now**

For RATINGS 2 through 8, please also rate EFFECTS:

**A**	**No concern**	**D**	**Some benefit**	**F**	**Don't Know**
**B**	**Some concern**	**E**	**Definitely a benefit**	**G**	**No opinion**
**C**	**Definitely a concern**				

****For "documented" RATINGS, please provide EVIDENCE (Attach to Survey)***

**INDUSTRY**		**ANIMAL**					
		**Dogs**		**Cats**		**Ferrets**	
		Rating	Effect	Rating	Effect	Rating	Effect
I	Cattle						
II	Swine						
III	Poultry						
IV	Fisheries						
V	Rabbits						
VI	Crops						
VII	Other: [list/rate]						

Comments: Please provide documentation, if possible.

ATTACH ADDITIONAL PAGES, AS NEEDED.

SeaSearch Biological Surveys

DOMESTIC CARNIVORE SURVEY

ACTIONS TAKEN **NOT APPLICABLE [ ]**

If unconfined animals are encountered in your jurisdiction, what ACTIONS would your Agency take?
Please check NOT APPLICABLE if this is not pertinent to your Agency or Jurisdiction.

Check ALL that apply: (X)

**ACTIONS**		**ANIMAL**		
		**Dogs**	**Cats**	**Ferrets**
I	None: not considered to be important			
II	None: desirable, but not feasible			
III	Live trap/take to shelter (assumed lost pet)			
IV	Live trap/euthanize			
V	Live trap/transport*			
VI	Take by any means			
VII	Refer to another agency*			
VIII	Attempt eradication			
IX	Unknown			

Comments: Please provide clarification and details, as necessary.

____

____

____

____

____

____

____

____

____

ATTACH ADDITIONAL PAGES, AS NEEDED.

SeaSearch Biological Surveys

DOMESTIC CARNIVORE SURVEY

ANIMAL CARE FACILITIES **NOT APPLICABLE [ ]**

Please list all ANIMAL CARE FACILITIES in your jurisdiction that accepts each type of animal. ***Please check NOT APPLICABLE if this is not pertinent to your Agency or Jurisdiction.***

Check ALL that apply:

**FACILITIES**		**ANIMAL**		
		**Dogs**	**Cats**	**Ferrets**
I	None - not considered to be important			
II	None - desirable, but not feasible			
III	Humane Shelters or similar			
IV	City/County Shelters			
V	Animal or Breed-specific Shelters/Rescues			
VI	Wildlife Rehabilitation, Refuges			
VII*	State/Provincial Agencies* [Please List Below]			
VIII	Unknown			

*Comments: Please provide clarification and details, as necessary.

____

____

____

____

____

____

____

____

____

____

ATTACH ADDITIONAL PAGES, AS NEEDED.

SeaSearch Biological Surveys

DOMESTIC CARNIVORE SURVEY

Do you have any SPECIAL CONCERNS regarding any of these three animals in your jurisdiction?

ATTACH ADDITIONAL PAGES, AS NEEDED.

A. URBAN / SUBURBAN AREAS (please describe):

B. RURAL / AGRICULTURAL AREAS (please describe):

C. PARKS / RECREATIONAL AREAS (please describe):

D. WILD LANDS / UNDEVELOPED AREAS (please describe):

Thank you for your participation.

PLEASE RETURN SURVEY AND ANY ATTACHMENTS.
